# The Influence of Plastic Processing on the Corrosion Resistance of the Alloy Based on the FeAl Intermetallic Phase After Long-Term Oxidation in Air Atmosphere

**DOI:** 10.3390/ma19081597

**Published:** 2026-04-15

**Authors:** Dorota Pasek, Janusz Cebulski, Maria Sozańska, Magdalena Popczyk, Jadwiga Gabor, Andrzej Swinarew

**Affiliations:** 1 Promobil S.C., Kopernika 12, 40-064 Katowice, Poland; 2 Department of Materials Technology, Faculty of Materials Engineering and Industrial Digitalization, Silesian University of Technology, Krasińskiego 8, 40-019 Katowice, Polandmaria.sozanska@polsl.pl (M.S.); 3 Faculty of Science and Technology, University of Silesia in Katowice, 75 Pułku Piechoty 1A, 41-500 Chorzów, Poland; 4 Department of Training and Nutrition in Sports, The Jerzy Kukuczka Academy of Physical Education, Mikołowska 72A, 40-065 Katowice, Poland

**Keywords:** oxidation, oxide layer, SEM, Al_2_O_3_, FeAl

## Abstract

**Highlights:**

- Formation of a continuous α-Al_2_O_3_ scale in Fe40Al5Cr0.2TiB at 700 °C in air.
- Plastic processing increases oxidation kinetics and mass gain vs. as-cast state.
- Oxide scale thickness remains submicrometric (~340 nm) after 2000 h.
- Microstructural changes modify diffusion paths and defect density.
- Plastic deformation affects kinetics without changing oxide phase composition.

**Abstract:**

This work presents a study of oxide-scale development on a B2 FeAl-based alloy (Fe40Al5Cr0.2TiB) during long-term isothermal oxidation at 700 °C in air, with particular emphasis on the effect of plastic processing. Oxidation tests were conducted for 300, 1000, and 2000 h. Surface morphology and chemical composition were examined using SEM/EDS, phase composition was identified by XRD, and local oxide thickness was measured by TEM. Surface topography was quantified by optical profilometry using Ra and Rz parameters. In both material states (as-cast and plastically processed), the alloy formed a continuous α-Al_2_O_3_-based scale. Plastic processing significantly affected oxidation kinetics, resulting in higher mass gain compared to the as-cast condition. Despite differences in mass gain, the average oxide-scale thickness after 2000 h remained in the submicrometric range (~340 nm) for both states. Surface topography analysis revealed differences in roughness and morphology associated with the material condition. The results demonstrate that plastic processing influences oxidation behavior primarily through microstructural modification, while the protective character of the alumina scale remains preserved. These findings provide data relevant to FeAl-based materials considered for high-temperature energy applications.

## 1. Introduction

Iron aluminide alloys based on the ordered B2 FeAl intermetallic phase remain attractive candidates for high-temperature service due to their relatively low density, the potential to form protective alumina scales, and favorable cost compared with conventional Ni-based superalloys [1,2,3]. For alumina-forming alloys, long-term corrosion resistance is controlled primarily by the selective oxidation of aluminum and the ability to establish a continuous Al_2_O_3_ layer that restricts further oxygen ingress and metal outward transport [2,3,4,5].

The protectiveness of thermally grown alumina scales depends not only on chemical composition but also on scale phase constitution, morphology, adherence, and growth kinetics [4,5,6,7]. During the initial stages of oxidation, transient alumina polymorphs (typically γ- and θ-Al_2_O_3_) may form prior to transformation into the thermodynamically stable α-Al_2_O_3_ phase [6,7,8,9]. The transient-to-α transformation is particularly important at intermediate temperatures, because it can influence the observed oxidation rate and scale morphology [8,9,10,11].

FeAl-based alloys, containing approximately 40 at.% Al, have been reported to develop alumina-based scales in air in the range of 700–1000 °C, commonly exhibiting parabolic kinetics once a protective layer is established [12,13,14]. However, oxidation behavior at ~700 °C is especially sensitive to exposure time and to the early-stage competition between transient oxides and the eventual development of α-Al_2_O_3_ [8,13]. Long-term studies extending to 1000–2000 h are therefore highly relevant for service-life assessment, because many engineering components (e.g., furnace fixtures, heat-exchange equipment, recuperators, boiler elements) operate for thousands of hours under quasi-steady thermal conditions [1,12,15,16].

In practical manufacturing, thermomechanical processing is routinely used to improve mechanical performance; however, it simultaneously modifies microstructure (grain size, defect density, internal stresses) and may therefore influence oxide nucleation, diffusion paths, and long-term scale growth [17,18]. This aspect is particularly important for ordered B2 intermetallics, where intrinsic point defects and their stability may affect diffusion processes relevant to oxidation [19,20]. In alumina-forming systems, microstructure-controlled effects have been demonstrated, including oxidation-mechanism changes associated with grain refinement and microstructural modification in Fe–Al-based alloys at 700 °C [21,22,23]. Grain boundaries and deformation-induced defects may act as fast diffusion paths, thereby influencing oxidation kinetics and scale development.

Despite the above progress, a quantitative, application-oriented comparison of long-term oxidation resistance in air for FeAl-based alloys in the as-cast versus plastically processed state—supported by mass-gain kinetics and post-exposure scale thickness—remains limited, especially at fixed intermediate temperature and extended exposure times. Therefore, the aim of the present study is to evaluate the influence of plastic processing on the long-term oxidation behavior of an Fe40Al5Cr0.2TiB alloy during isothermal exposure at 700 °C in laboratory air for 300, 1000, and 2000 h. Particular attention is devoted to oxidation kinetics based on mass-change measurements, oxide-scale morphology, phase composition of corrosion products, and oxide-layer thickness after prolonged exposure.

## 2. Materials and Methods

### 2.1. Materials for Research

The investigated material was an FeAl intermetallic-based alloy with the nominal composition Fe40Al5Cr0.2TiB (40.10 at.% Al, 4.86 at.% Cr, 0.18 at.% Ti, and 0.06 at.% B).

The alloy was prepared by vacuum induction melting using technically pure ARMCO iron, ARO aluminum (99.995% purity), aluminothermic chromium produced by the Kroll method, and amorphous boron. Melting was carried out in a Balzers VSG-2 vacuum induction furnace (Balzers, Lichtenstein).

In the as-cast condition, the alloy exhibited chemical segregation typical of Fe–Al intermetallic systems. To ensure chemical homogeneity, a homogenization heat treatment was applied. The alloy was annealed at 1050 °C for 72 h, which, according to previous investigations, enables effective elimination of microsegregation and results in a uniform microstructure. This treatment ensured chemical homogeneity and minimized microsegregation prior to further processing and testing.

After homogenization, the alloy was plastically processed by co-directional extrusion. The starting material was a cylindrical cast billet with a diameter of 22.2 mm. Extrusion was initiated at 1100 °C and carried out in a single-pass process. After extrusion, the diameter of the material was reduced to 15.7 mm, corresponding to an extrusion ratio of approximately 2:1.

The applied thermomechanical treatment enabled the production of crack-free extruded material suitable for further oxidation testing.

The extrusion ratio (λ) was determined as the ratio of the initial cross-sectional area of the billet to the final cross-sectional area after extrusion. For cylindrical specimens, this relation can be expressed as:

$$\lambda ={\left(\frac{D_{0}}{D_{f}}\right)}^{2}$$

where

D_0_—initial billet diameter;

Df—final diameter after extrusion.

For $D_{0}=22.2$ mm and $D_{f}=15.7$ mm, this gives an extrusion ratio of approximately 2:1.

The true strain (ε) imposed during extrusion was calculated assuming volume constancy during plastic deformation:

ε=ln λ

which is equivalent to:

$$\varepsilon =2ln\left(\frac{D_{0}}{D_{f}}\right)$$

For the applied extrusion conditions, the true strain was: ε≈0.69.

Physically, the true strain represents the logarithmic measure of plastic deformation and reflects the intensity of microstructural modification introduced during the extrusion process. In intermetallic FeAl-based alloys, such deformation may significantly influence defect density, substructure formation, and diffusion paths, all of which are relevant to subsequent high-temperature oxidation behavior.

### 2.2. Experimental Procedure

Cylindrical specimens for oxidation tests were prepared from the Fe40Al5Cr0.2TiB alloy in both the as-cast and plastically processed conditions. The samples had diameters of 5.5 or 6.0 mm and heights ranging from 1.3 to 3.0 mm, depending on the material condition. Due to the limited machinability of FeAl-based intermetallic alloys, the specimens were sectioned using electrical discharge machining (EDM) to avoid cracking and mechanical damage. Prior to oxidation, the specimen surfaces were ground using SiC abrasive papers (Struers, Ballerup, Denmark) with grit sizes of 120 and 600. After grinding, the samples were ultrasonically cleaned in acetone and dried. The initial mass of each specimen was measured using a RADWAG WAA100/C/1 analytical balance (Radom, Poland) with a measurement accuracy of 1 × 10^−4^ g. Isothermal oxidation tests were carried out at 700 °C in laboratory air. The specimens were placed in ceramic crucibles and introduced into the hot zone of a laboratory furnace designed for long-term oxidation experiments. Separate specimens were used for each exposure time. After 300 h, 1000 h, and 2000 h of oxidation, the corresponding samples were removed from the furnace, cooled in air to room temperature, and weighed. The mass change was determined by comparing the post-exposure mass with the initial mass. The specific mass change was calculated by dividing the measured mass gain by the initial surface area of each specimen and expressed in mg·cm^−2^.

The microstructure was examined using light optical microscopy (LOM) with a Olympus GX51 microscope (Olympus Corporation, Tokyo, Japan).

After oxidation, the morphology of the oxide scales was examined using scanning electron microscopy (SEM). Observations of specimens oxidized for 300, 1000, and 2000 h were performed using Hitachi S-3400N and Hitachi S-4200 microscopes (Hitachi High-Tech Corp., Tokyo, Japan) equipped with secondary electron (SE) and backscattered electron (BSE) detectors.

The chemical composition of the oxidation products was analyzed by energy-dispersive X-ray spectroscopy (EDS) using a Thermo Noran System Six spectrometer (Waltham, MA, USA) coupled with the Hitachi S-3400N microscope. The EDS measurements were performed at an accelerating voltage of 15 kV.

Phase identification of the oxide scales was conducted by X-ray diffraction (XRD). XRD analyses were performed for specimens oxidized for 2000 h, for which the oxide-scale thickness allowed for the reliable identification of crystalline phases. For shorter oxidation times, due to the limited thickness of the oxide scales, phase identification was supported primarily by morphological observations and comparison with literature data.

Surface topography and roughness parameters of the oxide scales were determined using a non-contact optical profilometer (MicroProf 3000, FRT GmbH, Bergisch Gladbach, Germany) equipped with a white-light interferometric head. Based on the measured surface profiles, the roughness parameters Ra and Rz were calculated to quantitatively evaluate changes in surface topography as a function of oxidation time.

## 3. Results

### 3.1. Material Characteristics

**Figure 1:**
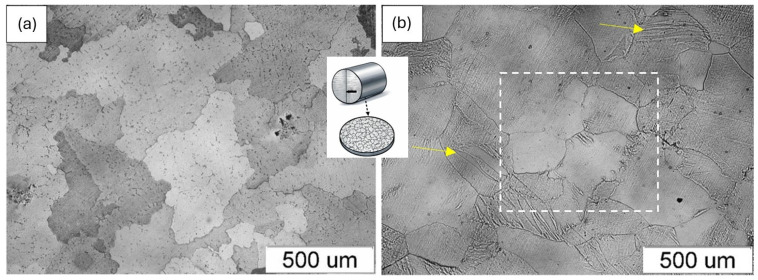
Cross-sectional microstructure of the Fe40Al5Cr0.2TiB alloy prior to oxidation: (**a**) as-cast condition; (**b**) after plastic processing (extrusion). The yellow arrows indicate regions within the grains where microstructural changes induced by plastic processing are observed, while the white rectangle highlights an area where grain refinement is particularly visible.

Prior to oxidation testing, the microstructure of the Fe40Al5Cr0.2TiB alloy was characterized in both the as-cast and plastically processed conditions. The results obtained by light microscopy are presented in Figure 1. The alloy in the as-cast condition exhibited a coarse-grained microstructure with heterogeneous grain-size characteristics and irregular grain-boundary morphology (Figure 1a). The grains varied significantly in size, and casting-related structural heterogeneities were observed. Plastic processing by extrusion resulted in measurable microstructural modification (Figure 1b). More uniform grain-size characteristics were observed, accompanied by a reduction in casting-related defects. The grains exhibited a more regular morphology, and deformation-induced features characteristic of extrusion processing were visible (marked with an arrow in Figure 1b).

It should be noted that the above observations are based on qualitative microstructural examinations performed on representative cross-sectional areas of the investigated samples, which consistently indicated differences between the analyzed conditions.

**Figure 2:**
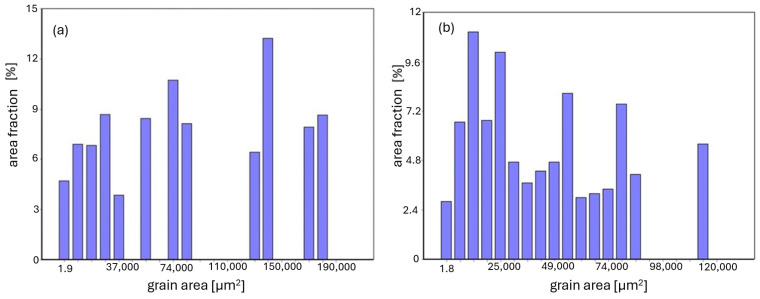
Grain-size characteristics of the Fe40Al5Cr0.2TiB alloy before oxidation, expressed as surface fraction versus planar grain cross-sectional area, based on measurements from representative cross-sectional areas (non-statistical data): (**a**) as-cast condition; (**b**) after plastic processing.

The grain-size characteristics, expressed as the surface fraction as a function of the planar grain cross-sectional area, are presented in Figure 2. The presented data illustrate differences in grain-size characteristics between both material states. A tendency toward grain refinement after plastic processing can be observed. However, this effect is relatively moderate. The presented plots should not be interpreted as statistically representative grain size distributions. These data are based on measurements from representative areas and are intended for comparative purposes only.

For the as-cast alloy, the average planar grain cross-sectional area was 0.027 mm^2^, while after plastic processing it decreased to 0.022 mm^2^. This difference suggests a tendency toward grain refinement; however, it should be interpreted with caution due to the limited statistical representativeness of the analysis.

For quantitative evaluation, representative micrographs were selected for each condition. Therefore, the obtained grain size parameters should be treated as indicative and comparative, rather than statistically representative.

It should also be emphasized that the investigated alloy exhibits limited plastic deformability, and the applied degree of deformation was relatively low. Consequently, the observed grain refinement is not very pronounced. Nevertheless, the applied plastic processing led to measurable improvements in mechanical properties, accompanied by noticeable changes in the microstructure.

### 3.2. Oxidation Kinetics

**Figure 3:**
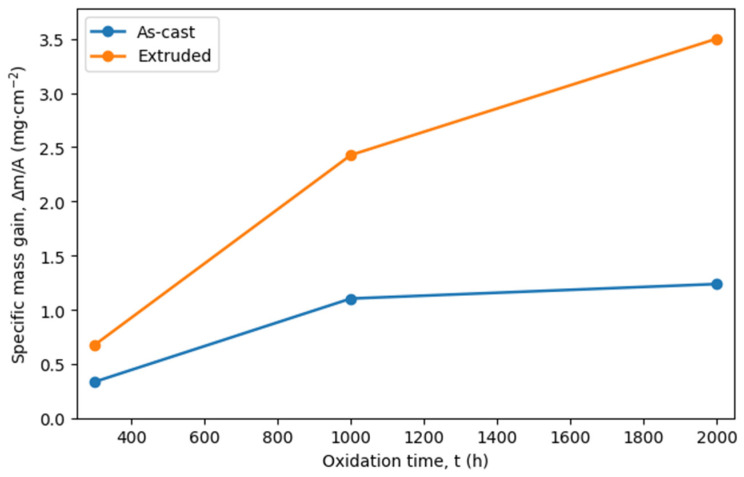
Specific mass gain (Δm/A) as a function of oxidation time for the Fe40Al5Cr0.2TiB alloy in the as-cast and extruded conditions during isothermal exposure at 700 °C in air. Each data point represents the mean value obtained from three specimens.

The oxidation kinetics of the Fe40Al5Cr0.2TiB alloy in the as-cast and extruded conditions at 700 °C are presented in Figure 3. The results are expressed as specific mass gain (Δm/A), calculated with respect to the initial surface area of each specimen.

For the as-cast material, the mass gain increased from 0.335 mg·cm^−2^ after 300 h to 1.103 mg·cm^−2^ after 1000 h and reached 1.237 mg·cm^−2^ after 2000 h of exposure. A noticeable reduction in the mass-gain increment between 1000 h and 2000 h suggests a tendency toward oxidation-rate stabilization during long-term exposure.

In contrast, the extruded material exhibited significantly higher mass gains at all exposure times. The values increased from 0.677 mg·cm^−2^ after 300 h to 2.428 mg·cm^−2^ after 1000 h and further to 3.501 mg·cm^−2^ after 2000 h. Unlike the as-cast condition, no clear tendency toward stabilization was observed within the investigated time range.

The results indicate that plastic processing markedly affected the long-term oxidation behavior of the alloy, resulting in accelerated mass gain under the applied test conditions.

### 3.3. Surface Morphology After Oxidation in Air

**Figure 4:**
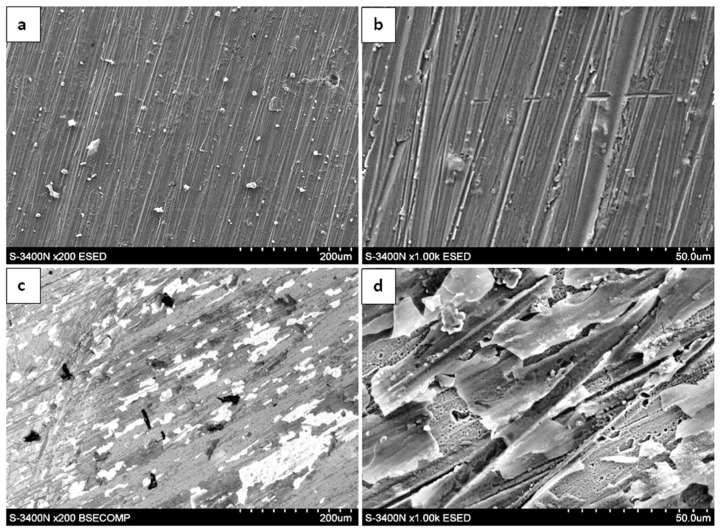
Surface of Fe40Al5Cr0.2TiB alloy oxidized for 300 h in air: (**a**,**b**)—as cast; (**c**,**d**)—after plastic processing.

**Figure 5:**
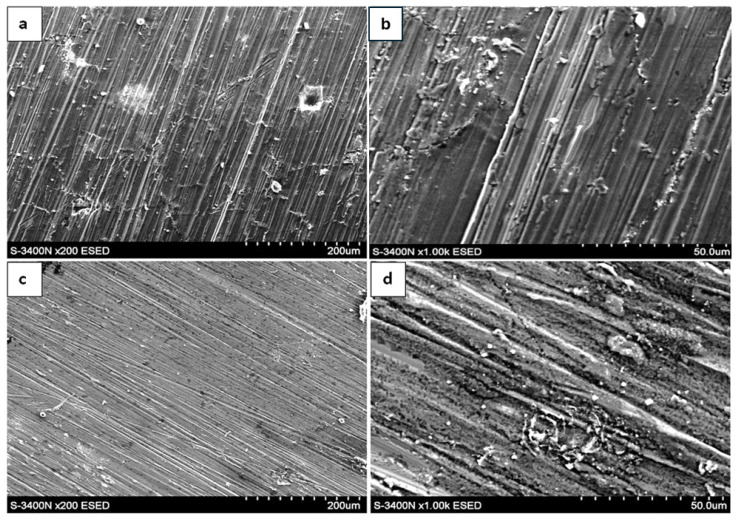
Surface of Fe40Al5Cr0.2TiB alloy oxidized for 1000 h in air: (**a**,**b**)—as cast; (**c**,**d**)—after plastic processing.

**Figure 6:**
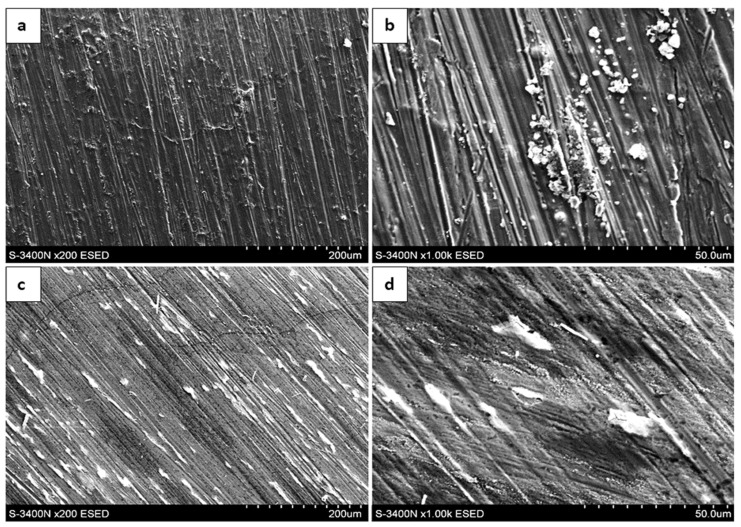
Surface of Fe40Al5Cr0.2TiB alloy oxidized for 2000 h in air: (**a**,**b**)—as cast; (**c**,**d**)—after plastic processing.

The surface morphology of the oxide scales formed after 300, 1000, and 2000 h of exposure at 700 °C in laboratory air is presented in Figure 4, Figure 5 and Figure 6. SEM observations were carried out using both an environmental secondary electron detector (ESED) and a backscattered electron detector operating in compositional mode (BSECOMP). The ESED detector was used to obtain topographical contrast of the surface, while the BSE detector provided compositional contrast, allowing for differentiation between regions of varying chemical composition within the oxide scale.

After 300 h of oxidation, the surface of the as-cast material was covered by a relatively continuous oxide layer. The scale exhibited locally heterogeneous morphology, with areas of different contrast visible in BSE mode. These contrast variations may be associated with compositional differences; however, morphological effects cannot be excluded.

After 1000 h of exposure, the oxide layer appears relatively more continuous; however, local porosity and heterogeneities are still present. The observed surface morphology may suggest thickening of the scale, although this assessment is based on qualitative SEM observations. No extensive spallation was observed.

After 2000 h of oxidation, the oxide scale appears more uniform. However, this observation should be treated as qualitative. The morphology suggests the development of a relatively protective oxide layer, which is consistent with the reduced mass-gain increment observed at prolonged exposure times.

In contrast, the extruded material exhibited a different oxidation morphology. After 300 h, the oxide layer was more developed compared to the as-cast state. The surface showed pronounced heterogeneity, and localized features such as nodular growth or surface irregularities were observed.

After 1000 h, the surface morphology indicates increased roughness and complexity. The oxide layer appears less uniform, which may suggest more intensive oxide growth; however, this interpretation remains qualitative.

After 2000 h, the surface was covered by a thick oxide scale characterized by heterogeneous features. No clear indication of oxidation-rate stabilization can be inferred solely from the morphological observations.

**Figure 7:**
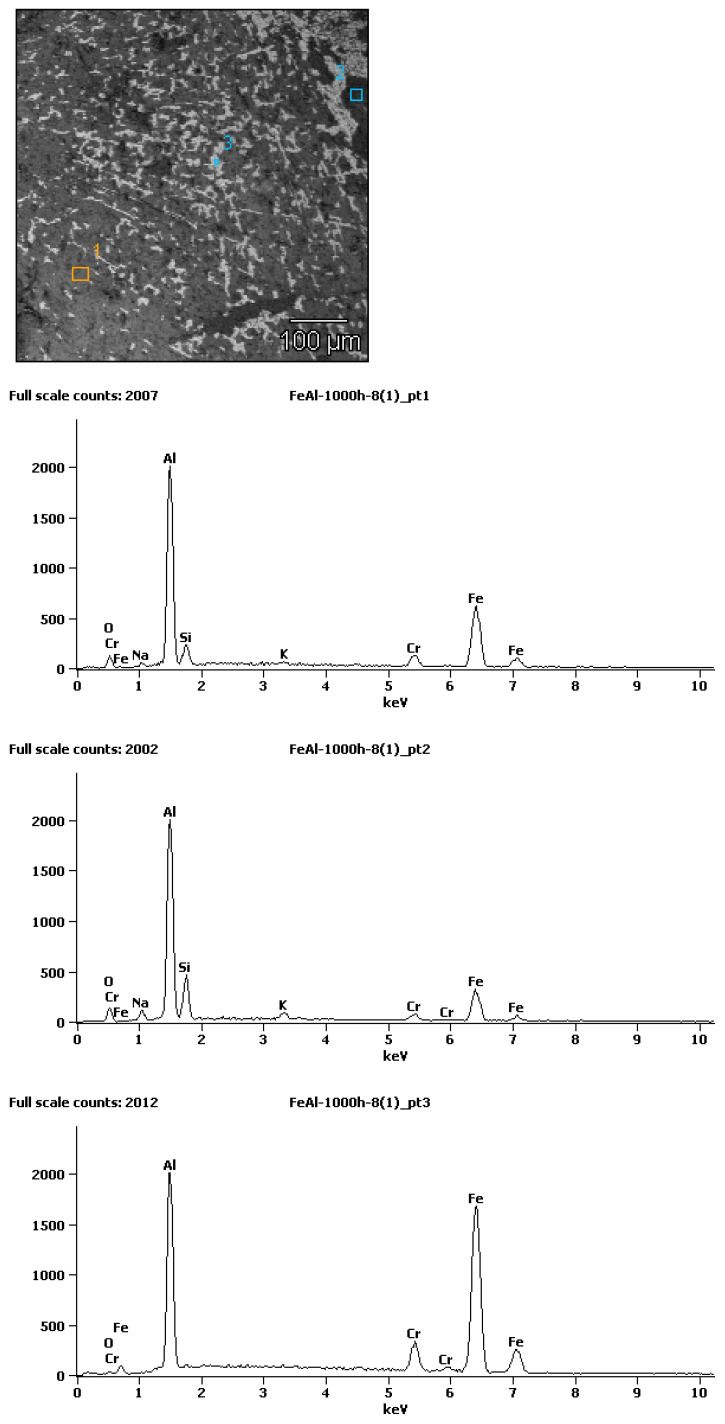
SEM image of the oxide scale formed after 1000 h of oxidation at 700 °C after plastic processing, with marked analysis points, together with a representative EDS spectrum obtained from the selected area, showing the elemental composition of the oxidation products.

To support the interpretation of the observed contrast variations and the chemical nature of the oxidation products, a representative EDS spectrum was acquired from selected areas of the oxide scale (Figure 7). The spectrum confirms the presence of elements associated with the oxide layer, including aluminum and oxygen, as well as contributions from the substrate.

### 3.4. Surface Topography and Roughness

**Figure 8:**
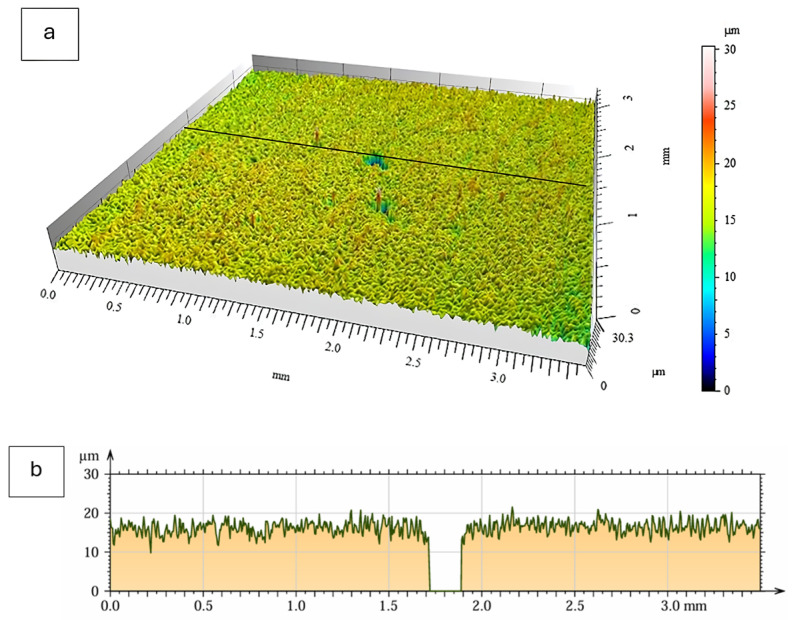
Surface topography of the Fe40Al5Cr0.2TiB alloy in the as-cast condition after 2000 h of oxidation at 700 °C in air: (**a**) Three-dimensional surface map obtained by optical profilometry; (**b**) surface profile along the selected measurement line.

**Figure 9:**
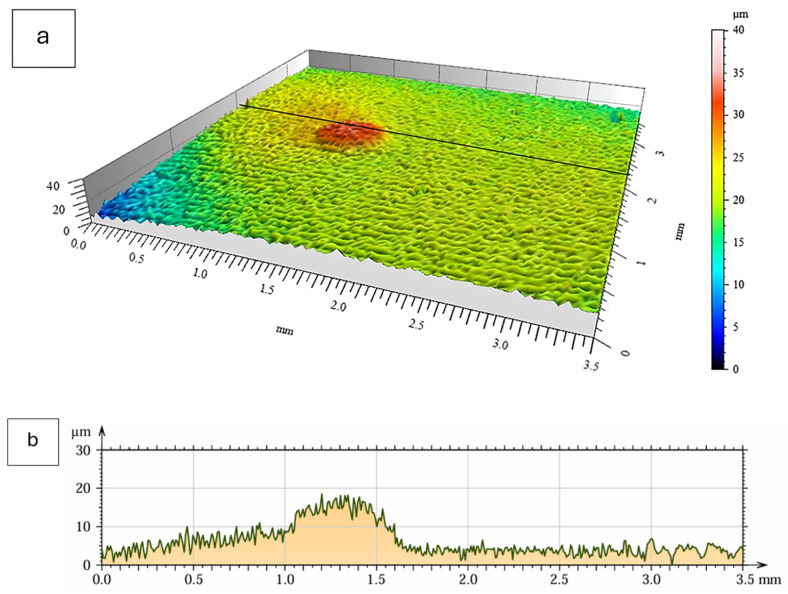
Surface topography of the Fe40Al5Cr0.2TiB alloy in the extruded condition after 2000 h of oxidation at 700 °C in laboratory air: (**a**) Three-dimensional surface map obtained by optical profilometry; (**b**) surface profile along the selected measurement line.

Surface topography of the oxide scales formed after 2000 h of oxidation at 700 °C was analyzed using non-contact optical profilometry (MicroProf 3000, FRT GmbH, Bergisch Gladbach, Germany). Representative three-dimensional surface maps and corresponding line profiles for both material states are presented in Figure 8 and Figure 9. The use of Ra and Rz parameters is also justified by their relevance in tribological applications, where surface roughness significantly affects friction behavior, contact stresses, and wear performance.

The oxide scale formed on the as-cast alloy exhibited a moderately developed surface morphology characterized by irregular but relatively uniformly distributed height variations. The surface profile showed continuous fluctuations without sharp local protrusions. The measured roughness parameters were Ra = 1.12 ± 0.15 µm and Rz = 7.75 ± 0.65 µm.

In contrast, the extruded alloy displayed a generally smoother background surface, although a localized elevation feature was observed in the analyzed area. Apart from this isolated region, the surface relief appeared more uniform compared to the as-cast condition. The corresponding roughness values were Ra = 0.65 ± 0.11 µm and Rz = 5.06 ± 0.55 µm.

Despite the higher overall mass gain observed for the extruded material, the profilometric measurements indicate a lower average roughness after long-term oxidation. The surface topography of the oxide scale formed on the extruded alloy can therefore be described as more homogeneous on the analyzed length scale.

### 3.5. Phase Composition of the Oxide Scales

**Figure 10:**
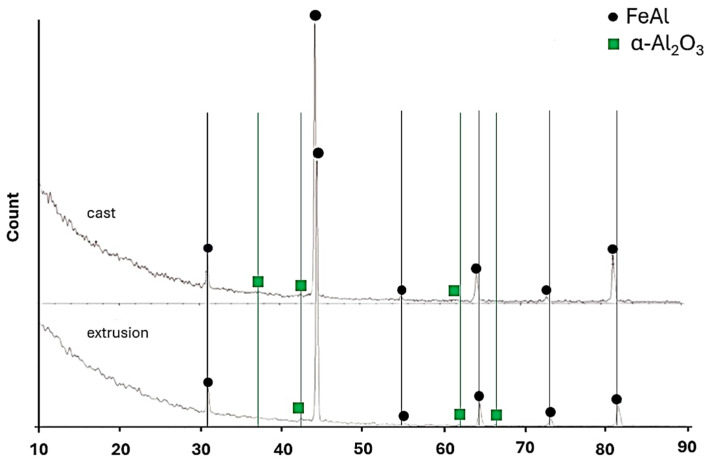
X-ray phase analysis of oxidation products formed on the surface of the Fe40Al5Cr0.2TiB alloy after corrosion tests in air for 2000 h.

The phase composition of the oxide scales formed after 2000 h of oxidation at 700 °C was analyzed by X-ray diffraction (XRD). The diffraction patterns obtained for both the as-cast and extruded conditions are presented in Figure 10.

In both material states, the dominant diffraction peaks correspond to the FeAl intermetallic phase originating from the substrate. In addition to the substrate reflections, characteristic peaks of α-Al_2_O_3_ were identified, confirming the formation of aluminum oxide during long-term exposure.

No additional crystalline iron oxide phases were detected within the resolution limits of the applied method. The presence of intense FeAl reflections indicates that the oxide scale remained relatively thin after 2000 h of oxidation, allowing for partial penetration of the X-ray beam into the substrate.

The diffraction patterns obtained for the as-cast and extruded materials were qualitatively similar, indicating that plastic processing did not alter the phase composition of the oxide scale formed under the applied conditions. The oxide layer in both cases consisted predominantly of thermodynamically stable α-Al_2_O_3_.

### 3.6. Cross-Sectional Analysis

**Figure 11:**
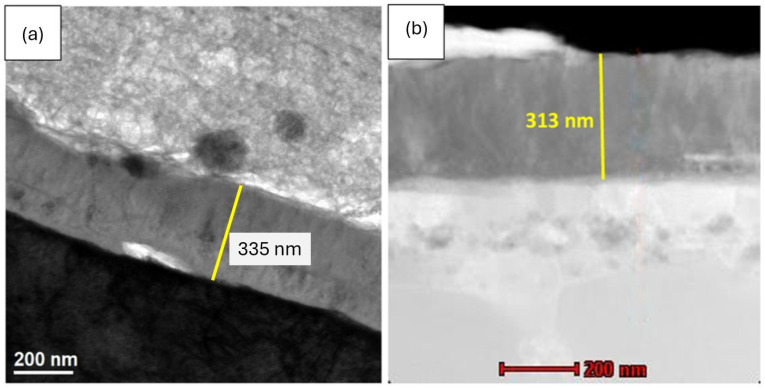
Thickness of the oxide layer of the oxidized Fe40Al5Cr0.2TiB alloy during 2000 h: (**a**)—after casting, (**b**)—after plastic working.

The thickness of the oxide scales formed after 2000 h of oxidation at 700 °C was evaluated using transmission electron microscopy (TEM) (Titan 80–300, FEI Company, Hillsboro, OR, USA). Cross-sectional micrographs are shown in Figure 11. Due to the limited observation area inherent to TEM analysis, the measured oxide thickness should be regarded as local. The oxide-layer thickness varies depending on the analyzed region. For the as-cast alloy oxidized in air, the average oxide-scale thickness determined from five measurements was approximately 340 ± 82 nm. The relatively high standard deviation indicates local thickness variations within the analyzed area. For the plastically processed alloy, the average oxide-scale thickness determined from five measurements was approximately 347 ± 39 nm, indicating a more uniform oxide-layer thickness compared to the as-cast condition. In both material states, the oxide layer appeared continuous and compact at the nanoscale, consistent with the formation of a thin protective alumina scale.

## 4. Discussion

The results obtained in the present study demonstrate that plastic processing modifies the oxidation behavior of the Fe40Al5Cr0.2TiB alloy during long-term exposure at 700 °C in laboratory air. Although both material states formed predominantly α-Al_2_O_3_, as confirmed by XRD analysis, clear differences in oxidation kinetics were observed. The extruded material exhibited consistently higher specific mass gain compared to the as-cast condition throughout the entire exposure period. This indicates that plastic deformation influences the oxidation rate without altering the fundamental phase composition of the oxide scale.

Microstructural characterization prior to oxidation revealed moderate grain refinement and increased structural uniformity after extrusion. While grain refinement may lead to an increased density of grain boundaries, which are often considered fast diffusion paths, the relatively small difference in grain size suggests that this factor alone cannot explain the observed differences in oxidation kinetics. Literature data indicate that the influence of grain size on oxidation behavior is not unambiguous and depends on the dominant diffusion mechanisms and specific material system [24,25].

In addition to grain size, plastic processing modifies the distribution of casting-related heterogeneities and the spatial arrangement of microstructural features. These changes may influence the distribution of oxidation nucleation sites and the uniformity of oxide growth. In the as-cast material, local heterogeneities may act as preferential sites for oxidation, leading to localized oxide growth and increased morphological variability. In contrast, the more uniform microstructure of the extruded material may promote a more homogeneous distribution of oxidation sites.

It should be noted that the specific mass gain reflects the overall oxygen uptake of the specimen, whereas the oxide-scale thickness determined by TEM represents a local measurement. Therefore, these parameters are not expected to correlate directly. In the present case, the higher mass gain observed for the plastically processed material indicates intensified overall oxidation processes, while the comparable local oxide thickness suggests that scale growth may proceed in a more spatially uniform manner.

This apparent discrepancy between global oxidation kinetics and local scale thickness highlights the importance of considering both microstructural features and measurement scale when interpreting oxidation behavior. The results suggest that plastic deformation modifies not only diffusion pathways but also the spatial distribution of oxide growth, leading to differences in mass gain without a proportional increase in local thickness.

The combination of higher mass gain and lower surface roughness observed for the plastically processed material indicates that oxide-scale growth is influenced not only by diffusion kinetics but also by the uniformity of the growth process. The lower roughness values together with the smaller variation in oxide-scale thickness revealed by TEM suggest the formation of a more homogeneous oxide layer. This behavior may be associated with microstructural modifications induced by plastic processing.

The potential role of deformation-induced defects, including dislocations, is considered in the context of diffusion processes; however, their contribution was not directly evaluated in the present study and should therefore be treated as a possible, rather than a confirmed, mechanism [26].

Overall, the obtained results indicate that oxidation behavior is governed by a combination of microstructural factors rather than a single dominant parameter.

## 5. Conclusions

The influence of plastic deformation on the oxidation behavior of the Fe40Al5Cr0.2TiB alloy during long-term exposure at 700 °C in air was investigated. Based on the obtained results, the following conclusions can be drawn:

1. Both the as-cast and plastically processed alloy formed a continuous and adherent α-Al_2_O_3_ scale during oxidation at 700 °C, confirming the alumina-forming character of the alloy.
2. Plastic processing significantly modified the oxidation kinetics, resulting in a higher specific mass gain compared to the as-cast condition.
3. Despite differences in mass gain, the average oxide-scale thickness after 2000 h remained within the same order of magnitude for both states (≈340 nm).
4. The oxide scale exhibited good adherence in both material states; no cracking or spallation was observed.
5. Plastic deformation led to grain refinement and reduction in casting-related defects, which influenced oxidation behavior by modifying diffusion paths and promoting a more uniform distribution of oxidation sites across the surface.
6. The increased density of grain boundaries and deformation-induced defects enhanced diffusion processes, contributing to intensified oxidation kinetics without altering the phase composition of the oxide scale.
7. Overall, plastic deformation affects oxidation behavior primarily through microstructural modification, influencing both oxidation kinetics and oxide-scale growth characteristics, while the protective nature of the alumina scale remains preserved.

## Data Availability

The original contributions presented in this study are included in the article. Further inquiries can be directed to the corresponding authors.
